# Electronic Health Record Data Quality and Performance Assessments: Scoping Review

**DOI:** 10.2196/58130

**Published:** 2024-11-06

**Authors:** Yordan P Penev, Timothy R Buchanan, Matthew M Ruppert, Michelle Liu, Ramin Shekouhi, Ziyuan Guan, Jeremy Balch, Tezcan Ozrazgat-Baslanti, Benjamin Shickel, Tyler J Loftus, Azra Bihorac

**Affiliations:** 1 Department of Medicine, University of Florida, Gainesville, Florida, United States; 2 Intelligent Clinical Care Center, University of Florida, Gainesville, Florida, United States; 3 College of Medicine, University of Central Florida, Orlando, Florida, United States; 4 Department of Surgery, University of Florida, Gainesville, Florida, United States; 5 Department of Medicine, Division of Nephrology, Hypertension, and Renal Transplantation, University of Florida, PO Box 100224, Gainesville, Florida, 32610-0224, United States, 1 3522948580, 1 3523925365

**Keywords:** electronic health record, EHR, record, data quality, data performance, clinical informatics, performance, data science, synthesis, review methods, review methodology, search, scoping

## Abstract

**Background:**

Electronic health records (EHRs) have an enormous potential to advance medical research and practice through easily accessible and interpretable EHR-derived databases. Attainability of this potential is limited by issues with data quality (DQ) and performance assessment.

**Objective:**

This review aims to streamline the current best practices on EHR DQ and performance assessments as a replicable standard for researchers in the field.

**Methods:**

PubMed was systematically searched for original research articles assessing EHR DQ and performance from inception until May 7, 2023.

**Results:**

Our search yielded 26 original research articles. Most articles had 1 or more significant limitations, including incomplete or inconsistent reporting (n=6, 30%), poor replicability (n=5, 25%), and limited generalizability of results (n=5, 25%). Completeness (n=21, 81%), conformance (n=18, 69%), and plausibility (n=16, 62%) were the most cited indicators of DQ, while correctness or accuracy (n=14, 54%) was most cited for data performance, with context-specific supplementation by recency (n=7, 27%), fairness (n=6, 23%), stability (n=4, 15%), and shareability (n=2, 8%) assessments. Artificial intelligence–based techniques, including natural language data extraction, data imputation, and fairness algorithms, were demonstrated to play a rising role in improving both dataset quality and performance.

**Conclusions:**

This review highlights the need for incentivizing DQ and performance assessments and their standardization. The results suggest the usefulness of artificial intelligence–based techniques for enhancing DQ and performance to unlock the full potential of EHRs to improve medical research and practice.

## Introduction

The adoption of electronic health records (EHRs) optimistically promises easily searchable databases as an accessible means for prospective and retrospective research applications [1]. EHRs often fall short of these promises due to limited local data and poor data quality (DQ) [2,3]. To overcome these limitations, several institutions have harmonized databases and model ontologies, including PCORnet (The National Patient-Centered Clinical Research Network), All of Us, MIRACUM (Medical Informatics in Research and Care in University Medicine), and the EHDEN Project [4-7]. These programs strive to offer high-quality data for research purposes [2]. However, EHR DQ remains highly variable, with some studies showing completeness in EHR parameter values ranging from 60% to 100% [8,9]. Similar inconsistencies present a significant limitation to the generalizability and applicability of lessons learned across these datasets for broader medical and research purposes.

Multiple initiatives have aimed to measure and improve EHR data [10,11]. Early efforts in DQ assessment (DQA) demonstrated inconsistent reporting and a need for universal terminology standards in DQA efforts [11]. In response, attempts at a standardized ontology for DQA have been developed, such as through the efforts of the International Consortium for Health Outcomes Measurement, 3×3 DQA guidelines, and the terminologies proposed by Kahn et al [12] and Wang et al [8,12-15]. More recently, artificial intelligence (AI) and natural language processing techniques have automated quality initiatives, including data assessment and augmentation [16,17]. Nonetheless, these techniques introduce their own set of quality requirements, including fairness metrics, handling intolerable or lost data, and mitigating data drift [18]. Measuring the result of these techniques’ application in real-world clinical contexts has given rise to another field that has become crucial for EHR improvement, namely, data performance assessment (DPA) [19].

In this review, we critically evaluate peer-reviewed literature on the intersection of DQA and DPA applications, as well as trends in their automation [10-13,20-22]. The purpose of this scoping review was to combine the 3 to formulate a more clear road map for evaluating EHR datasets for medical research and practice.

## Methods

### Overview

This scoping literature review was conducted according to the 2018 PRISMA-ScR (Preferred Reporting Items for Systematic Reviews and Meta-Analyses extension for Scoping Reviews), whose checklist is shown in Checklist 1 [23].

### Literature Search

A search was performed for all full-text research articles published in English in PubMed from inception to May 7, 2023. A list of the exact search terms is included in Multimedia Appendix 1.

### Article Selection

Four investigators (JB, RS, TRB, and YPP) reviewed the selected studies during the title and abstract screening. Further 4 investigators (ML, RS, TOB, and YPP) conducted the full-text review and final extraction of articles. Title or abstract screening, full-text review, and final extraction were based on the consensus opinion between 2 independent reviewers. Conflicts were resolved by a third reviewer. Article management and calculations of interrater reliability and Cohen κ were performed using Covidence systematic review software (Veritas Health Innovation).

### Inclusion Criteria

Titles and abstracts were screened to include original research articles assessing the DQ and performance of all or part of a hospital’s EHR system. We looked for studies reporting on 1 or more aspects of DQ (the assessment of EHR data without consideration of follow-up actions) and data performance (the assessment of EHR data applications) as defined (Table 1).

**Table 1.** Data quality and performance indicator definitions, mitigation strategies, and references.

	Definition	Mitigation strategies	Relevant studies
**Data quality**			
Completeness (or, conversely, missingness)	The absence of data points, without reference to data type or plausibility [12]	Automated data extraction; data imputation	[2-6,8,9,24-37]
Conformance	The compliance of data with expected formatting, relational, or absolute definitions [12]	Preemptively enforced data format standardization	[2-6,8,14,24-27,29-33,36,38]
Plausibility	The possibility that a value is true given the context of other variables or temporal sequences (ie, patient date of birth must precede date of treatment or diagnosis) [12]	Periodic realignment with logic rule sets or objective truth standards; thresholding	[4-6,8,14,25,27,28,30-33,35,37-39]
Uniqueness	The lack of duplicate data among other patient records [8]	Two-level encounter or visit data structure	[8]
**Data performance**			
Correctness or accuracy	Whether patient records are free from errors or inconsistencies when the information provided in them is true [10,13]	Periodic validation against internal and external gold standards	[2,7-9,14,23,24,28]
Currency or recency	Whether data were entered into the EHR^a^ within a clinically relevant time frame and is representative of the patient state at a given time of interest [10,13]	Enforcing predetermined hard and soft rule sets for timeline of data entry	[2,4,9,27,32,34,36]
Fairness (or, conversely, bias)	The degree to which data collection, augmentation, and application are free from unwarranted over- or underrepresentation of individual data elements or characteristics	Periodic review against a predetermined internal gold standard or bias criterion	[3,19,22,24,27,35]
Stability (or, conversely, temporal variability)	Whether temporally dependent variables change according to predefined expectations [10,12]	Periodic measurement of data drift against a baseline standard of data distribution	[4,8,19,31]
Shareability	Whether data can be shared directly, easily, and with no information loss [3]	Preemptively enforced data standardization	[2,3]
Robustness	The percent of patient records with tolerable (eg, inaccurate, inconsistent, and outdated information) versus intolerable (eg, missing required information) data quality problems [24]	Timely identification of critical data quality issues	[24]

a EHR: electronic health record.

### Data Quality

#### Conformance

*Conformance* refers to the compliance of data with expected formatting, relational, or absolute definitions [12].

#### Plausibility

*Plausibility* refers to the possibility that a value is true given the context of other variables or temporal sequences (ie, the patient’s date of birth must precede the date of treatment or diagnosis) [12].

#### Uniqueness

*Uniqueness* refers to the lack of duplicated records [8].

#### Completeness (or Conversely, Missingness)

With regard to completeness, *missingness* is the absence of requested data points, without reference to conformance or plausibility as defined [12].

### Data Performance

#### Correctness or Accuracy

*Correctness* or *accuracy* refers to whether patient records are free from errors or inconsistencies when the information provided in them is true [10,13].

#### Currency or Recency

*Currency* or *recency* refers to whether data were entered into the EHR within a clinically relevant time frame and are representative of the patient state at a given time of interest [10,13].

#### Fairness (or Conversely, Bias)

With regard to bias, *fairness* refers to the degree to which data collection, augmentation, and application are free from unwarranted over- or underrepresentation of individual data elements or characteristics.

#### Stability (or Conversely, Temporal Variability)

With regard to stability, *temporal variability* refers to whether temporally dependent variables change according to predefined expectations [10,12].

#### Shareability

*Shareability* refers to whether data can be shared directly, easily, and with no information loss [3].

#### Robustness

*Robustness* refers to the percent of patient records with tolerable (eg, inaccurate, inconsistent, and outdated information) versus intolerable (eg, missing required information) DQ problems [24].

We additionally included studies reporting on data imputation methods, defined as techniques used to fill in missing values in an EHR, such as through statistical approximation and the application of AI.

### Exclusion Criteria

We excluded tangential analyses of DQ in articles focused primarily on clinical outcomes. As such, studies discussing data cleaning as part of quantifying clinical outcomes were excluded from our analysis. Proposals or study protocols with no results were also excluded during the screening process.

### Article Quality Assessment

Full-text articles were additionally scored as having or missing the criteria for (1) data integrity: comprehensiveness for each main outcome, including attrition and exclusions from the analysis and reasons for them; (2) method clarity: a clear description of DQA data sources, analysis steps, and criteria; (3) outcome clarity: outcomes reporting in plain language, in their entirety, and without evidence for selective reporting; and (4) generalizability: applicability of DQ techniques described in the article to other clinical settings.

## Results

### Article Characteristics

**Figure 1.**
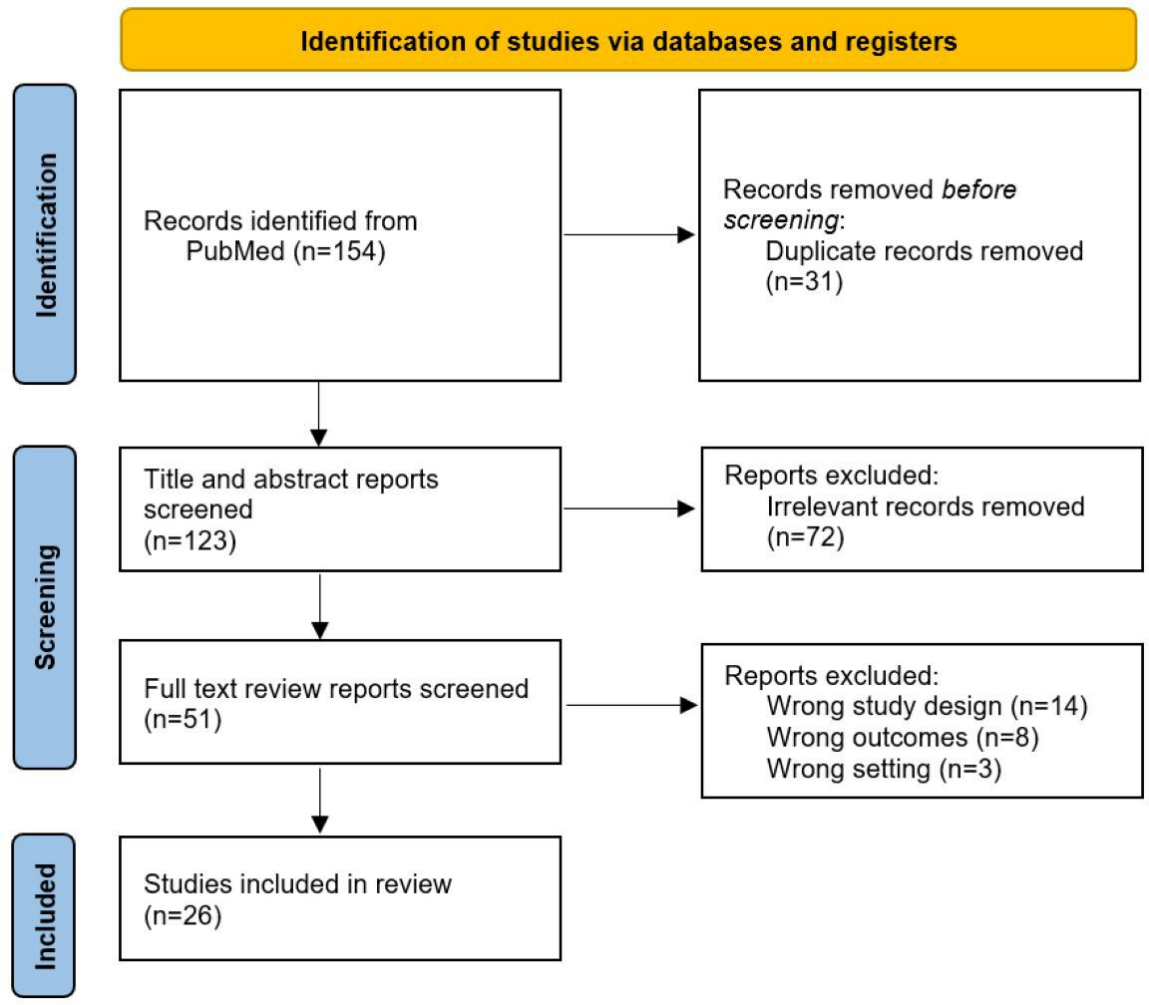
PRISMA 2020 flow diagram detailing study selection and reasons for exclusion for all articles considered for this scoping review. PRISMA: Preferred Reporting Items for Systematic Reviews and Meta-Analyses.

The flow diagram for article selection is shown in Figure 1. A total of 154 records were identified using the search terms defined in Multimedia Appendix 1 using the PubMed library. After the removal of 31 duplicates and the 72 articles identified as irrelevant, 51 studies proceeded to full-text review. Full-text review excluded a further 25 articles owing to reasons listed in Figure 1, leaving a final total of 26 original research studies [2-6,8,9,14,19,22,24-39]. The Cohen κ between the different pairs of reviewers ranged from 0.28 to 0.54 during the screening process and from 0.54 to 1.00 during the full-text review.

Study characteristics are shown in Table 2 and Multimedia Appendix 2. Exactly half of the identified articles targeted general EHR DQ analysis [4-6,19,22,27-32,38,39], while the other half focused on a particular specialty or diagnosis (Table 2) [2,3,8,9,14,24-26,33-37]. The latter included primary care (n=3, 12%) [35-37], cardiovascular disease (n=3, 12%) [8,33,34], anesthesia or pain medicine (n=2, 8%) [14,26], intensive care units (n=2, 8%) [3,25], and pediatrics [24], oncology [2], and infectious disease (n=1 each, 4%) [9].

Article quality assessment conducted as part of our review process identified 14 (54%) of the articles [2-6,8,9,19,22,24-36,38,39] had at least 1 common study design or reporting limitation, with 5 of the articles having more than 1 [14,24,33,36,38]. Among these, 6 (30% of all errors) articles did not clearly state their methods [3,27,28,33,36,39], 5 (25%) had incomplete data [24,29,33,36,38], 5 were not generalizable to other settings [4,24-26,33], and 4 did not clearly state their outcomes (Table 2) [31,34,38,39].

Commonly referenced DQ and performance indicators are summarized in Table 3. Respective definitions, mitigation strategies, and references are listed in Table 1.

**Table 2.** Frequency of clinical specialties among all papers and study limitations among all limitations identified by reviewers in this analysis.

Setting		Values, n (%)
**Specialty**		
	ICU^a^	2 (8)
	Anesthesia or pain med	2 (8)
	General	13 (50)
	Cardiovascular	3 (12)
	Infectious disease	1 (4)
	Oncology	1 (4)
	Pain medicine	0 (0)
	Pediatrics	1 (4)
	Primary care	3 (12)
**Limitations**		
	Incomplete data	5 (25)
	Methods not clearly stated	6 (30)
	Outcomes not clearly stated	4 (20)
	Not generalizable to other settings	5 (25)

a ICU: intensive care unit.

**Table 3.** Elements of data quality and performance commonly referenced by papers included in this review.

Data Quality and Performance Element		Values, n (%)
**Data quality**		
	Completeness	21 (81)
	Conformance	18 (69)
	Plausibility	16 (62)
	Uniqueness	1 (4)
**Data performance**		
	Correctness or accuracy	14 (54)
	Currency	7 (27)
	Fairness or bias	6 (23)
	Stability	4 (15)
	Shareability	2 (8)
	Robustness	1 (4)

### Data Quality Assessment

#### Completeness

Completeness was the most cited element of DQ analysis, with references in 21 (81%) of all articles [2-6,8,9,24-37]. Importantly, 19 (73%) studies integrated data from multiple clinical sites [2,4-6,9,19,22,24,26,30-39], which was associated with issues in data collection and missingness “across organizational structure, regulation, and data sourcing” [31]. Clinical domains reported to be prone to low data completeness included patient demographics, with Estiri et al [29] highlighting the issue for records of patient ethnicity and Thuraisingam et al [35] for mortality records (eg, missing year of death), and medication management, with Thuraisingam et al [35] highlighting the issue for dosage, strength, or frequency of prescriptions and Kiogou et al [34] for missing dates or reasons for discontinuation of medications.

To combat data missingness, Lee et al [22] used natural language processing algorithms to automatically extract data from patient records, while further 5 studies made use of data imputation techniques. Among the latter, 2 articles generated synthetic data, while another 3 supplemented datasets through information from external datasets. Fu et al [3] generated synthetic data by modeling providers’ assessments of EHR data based on different information sources according to their individual characteristics (eg, tendency to ascertain delirium status based on Confusion Assessment Method vs prior *International Statistical Classification of Diseases* coding or nursing flow sheet documentation), while Zhang et al [19] used a generative adversarial network (GAN) trained on real longitudinal EHR data to create single synthetic EHR episodes (eg, outpatient or inpatient visit). Meanwhile, Lee et al [33] supplemented existing EHR records on heart failure by aggregating data from open-source datasets of heart failure biomarkers (including the Database of Genotypes and Phenotypes and the Biologic Specimen and Data Repository Information Coordinating Center) and using literature guidelines to create a standard set of cardiovascular outcome measures, while Curtis et al [2] supplemented missing EHR mortality records with data from US Social Security Death Index and the National Death Index, and Mang et al [30] used a manually generated stand-alone synthetic dataset to test the development of a new software tool for DQ assessment.

#### Conformance

Conformance was the second most cited element of DQA, with references in 18 (69%) articles [2-6,8,14,24-27,29-33,36,38]. Similar to completeness*,* DQ checks on conformance were performed automatically across most studies. Mitigation strategies included enforcing strict formatting rules at the time of data entry, for example, by using *International Statistical Classification of Diseases* codes to define the cause of death or a diagnosis of delirium [2,3].

#### Plausibility

Plausibility was the third most cited element of DQA with references in 16 (62%) articles [4-6,8,14,25,27,28,30-33,35,37-39]. Clinical domains prone to issues with plausibility included patient baseline physical characteristics, medication, and laboratory records. Estiri et al [29] and Wang et al [39] reported significant rates of plausibility issues for baseline physical characteristics, with higher error rates for records of patient height as compared to weight, likely due to the multiple flow sheet fields for height, including “estimated,” “reported,” and “measured,” which are generally averaged or selectively dropped. Pharmacologic data were prone to issues with plausibility due to timeliness (eg, antiretroviral therapy was dispensed before or more than 30 days after the visit date [9]) or discrepancies between diagnoses and drugs (eg, nonsteroidal anti-inflammatory drug prescription on the date of gastroduodenal ulcer diagnosis [6]). Finally, laboratory results were also prone to issues with plausibility due to value ranges, units, timing (eg, laboratory time was at an invalid time of day or in the future), and discrepancies between diagnoses and laboratory records (eg, drug was documented as present but there was no laboratory record) or drug prescriptions and laboratory records (eg, metformin was prescribed prior to a documented hemoglobin A_1c _laboratory result, or warfarin was prescribed without a follow-up international normalized ratio laboratory result) [6]. Notably, this may reflect poorly integrated health care systems where laboratories are being drawn at disparate institutions.

A total of 18 (69%) studies used logic statements to assess plausibility [2,4-6,8,9,14,24,27,28,31-38], including rules to determine temporal plausibility (eg, laboratories drawn at an invalid time of day [eg, 10:65 AM] [6], extubation occurring prior to intubation [14], or death date occurring before birth date [32]), diagnostic or procedural plausibility (eg, a procedure marked as an outpatient when it is only performed on an inpatient basis [38] or an obstetric diagnosis given for a biologically male patient [6,9,38]), alignment with external standards or expectations (eg, laboratory result absent for diagnosis or drug [6] or demographic alignment of medication name and dose with expected value ranges [34]), and others. A total of 11 (42%) studies used thresholding to identify data of low or questionable quality [4,6,8,9,14,19,28,32,35,37,39], including clinical and physiological value ranges (eg, BMI between 12 and 90 kg/m^2^ [35] or fraction of inspired oxygen between 10% and 100% [14]) and logical thresholds (eg, recorded date of arrival prior to the date of data collection initiation [8] or difference of >730 days when comparing age in years and date of birth fields [9]).

#### Uniqueness

Finally, 1 (4%) study reported on data uniqueness. Aerts et al [8] measured the frequency of patient record duplications (ie, when patient records were erroneously copied during data merging or reprocessing). To reduce the rate of record duplications, the researchers in the study suggest a 2-level data structure, with more general patient data being recorded at the encounter level (which can include multiple visits during a single clinical episode) and diagnosis or procedure-specific data at the level of the particular visit.

### Data Performance Assessment

#### Correctness or Accuracy

Correctness or accuracy was the most cited element in data performance analysis, with references in 14 (54%) of all articles [2,8,9,14,19,25,26,32-37,39]. The metric was evaluated via manual review in 8 (57%) out of the 14 articles that reported the measure [2,8,14,25,26,34,36,39]. A total of 5 (36%) articles evaluated it in comparison to an external standard, including national registries [2,35], EHR case definitions based on billing codes [36], and literature guidelines with high research use [33], or, in the case of a newly proposed AI technique for synthetic data augmentation, comparison to a previously published GAN model performance [19]. A further 3 (21%) assessed correctness or accuracy against an internal standard by calculating the proportion of records satisfying internally predetermined rule sets [9,32,37]. Of note, Curtis et al [2] and Terry et al [36] used both manual review and comparison to an external gold standard for validation.

#### Currency or Recency

Recency was the second most cited data performance element, with references in 7 (27%) articles [2,4,9,27,32,34,36]. Among these, 5 (71%) studies evaluated the metric according to internally predetermined hard rule sets (eg, whether a patient who is obese had a weight recording within 1 year of the previous data point or whether data were entered into the EHR within 3 days of the clinical encounter [9,32,36]) or soft rule sets (eg, whether the data were entered into the EHR within a subjectively determined clinically actionable time limit [4,34]), while 2 (29%) used external standards, including national registries and guidelines [2,27].

#### Fairness or Bias

The third most cited data performance element was fairness or bias, with references in 6 (23%) articles [3,19,22,24,27,35]. Among these, Lee et al [22], Thuraisingam et al [35], Tian et al [27], and García-de-León-Chocano et al [24] assessed fairness by manual review, while Fu et al [3] and Zhang et al [19] did so through automated review against a predetermined internal gold standard (ie, distribution of data characteristics within a real EHR dataset) or data bias criterion (ie, critic model measuring Jensen-Shannon divergence between real and synthetic data over time), respectively.

#### Stability

Data stability was the fourth most cited performance element, referenced in 4 (15%) articles [4,8,19,31]. All 4 articles that measured data stability did so via temporal statistical analyses of data drift according to a predetermined internal baseline standard of data distribution [8,9,32,37].

#### Shareability

Shareability was referenced in 2 (8%) articles from our analysis [2,3]. Both studies measured the performance metric by way of manual review in a pre- and posttest analysis of data standardization [2,3].

#### Robustness

Finally, García-de-León-Chocano et al [24] reported on information robustness by way of statistical estimation of critical (eg, missing or null required values) versus noncritical (all other) DQ issues that may obstruct subsequent data applications and performance measures.

### Interventions for Improving DQ and Performance

Three articles included in our analysis reported effective interventions to improve DQ and performance [4,9,37]. In terms of DQ, Walker et al [37] reported an increase in compliance, with 155 completeness and plausibility data checks from 53% to 100% across 6 clinical sites after 3 rounds of DQA. In terms of DQ and performance, Puttkamer et al [9] reported both higher data completeness and recency following a continuous data reporting and feedback system implementation. Finally, Engel et al [4] reported increased shareability (concept success rate, ie, whether data partners converted information from their individual EHRs to the shared database)—an increase from 90% to 98.5%—and a notable reduction in the percentage of sites with over 3 DQ errors—a reduction from 67% to 35%—across 50+ clinical sites over 2 years.

## Discussion

### Principal Contributions and Comparison With Prior Work

This scoping review provides an overview of the most common and successful means of EHR DQ and performance analysis. The review adds to a growing body of literature on the subject, most recently supplemented by a systematic review by Lewis et al [40]. To our knowledge, ours is the first review of specialty-specific applications of DQ alongside performance assessments. We identified and analyzed a total of 26 original research articles recently published on the topic. The results serve to characterize the most common medical fields making use of such assessments, the methodologies they use for conducting them, and areas for specialty-specific, as well as generalizable, future improvement. Finally, the discussion proposes a set of 6 unique and practical recommendations for minimizing modifiable DQ and performance issues arising during data extraction and mapping.

### Article Characteristics

Our review noted a paucity of DQ assessments within clinical specialties, where expert domain knowledge plays a key role in identifying logic inconsistencies. Half of all identified articles concerned general EHR data assessments, while the other half focused on medical fields such as primary care, cardiovascular diseases, or intensive care unit or anesthesia, with the notable absence of psychiatry, emergency medicine, and any of the surgical specialties. This points to a lack of peer-reviewed research and underuse of DQ and performance strategies across a wide spectrum of the medical field. There is a wide knowledge gap between how data are entered and acted upon clinically and how they appear in silico. Therefore, more efforts need to be directed toward supporting EHR data assessment initiatives in these specialties, with close collaboration between clinical users and data scientists.

More than half of the articles included in this scoping review had common limitations, including using or reporting incomplete data, methods, and outcomes. Among the articles scoring high for incomplete data, the chief issues include data attrition during extraction [24,29] and unclear or missing reporting [33,36,38], pointing to a need for higher information interoperability and reporting standards, such as those put forth by Kahn et al [12]. These standards recommend using a harmonized and inclusive framework for the reporting of DQ assessments, including standardized definitions for completeness, conformance, plausibility, and other measures as discussed previously.

Similar issues were observed with methods reporting, with several articles underreporting steps in their data extraction or analysis, thereby limiting the replicability and generalizability of their findings [3,27,28,33]. Unclear reporting or underreporting was a substantial issue for outcomes as well, with low-scoring articles reporting only partial or too high-level results suggesting selective reporting bias [14,31,34,38]. To align with the standards set forth by articles scoring high in reporting quality, we recommend stating all data sourcing, methods, and results according to predetermined definitions of DQ or performance (see above) in enough detail such that they would be easily replicated by researchers at an unrelated institution.

A final article quality pitfall concerned articles that were too specific to a particular health system or clinical context. The chief issues among original research articles that in house scored “low” in our generalizability assessment concerned their overreliance on internal DQ checks or measures that could only be implemented within their specific institutional EHR [4,24-26,33]. To increase generalizability, we recommend relying on external DQ standards such as societal guidelines, previously published measures, or open-source databases, to the extent possible before resorting to the development of new in-house tools that impose limitations to generalizability outside the local clinical context [8,12-15].

### Data Quality Assessment

The marked drop-off between the use of completeness, conformance, and plausibility versus other indicators (Table 3) demonstrates that the field has settled on these measures as the main components of EHR DQ analysis. Taking this into consideration, we recommend measuring all 3 for a general assessment of clinical DQ. Of note, there is a significant drop-off between 81% (n=21) of studies reporting on completeness versus 69% (n=18) on conformance and 62% (n=16) on plausibility, which indicates an opportunity for limited but quick DQ “checks” using completeness measures only. More specialized analyses may require further reporting, including uniqueness in the event of data merger with the possibility of duplicate results. These may be particularly important in the case of EHR DQ assessments following information reconciliation from the merger of multiple data sources, including patient demographics or baseline physical characteristics and laboratory or pharmacological data, which were shown to be particularly prone to errors in DQ.

Our review additionally demonstrates that issues with data completeness, conformance, and plausibility may be at least partially addressed with data imputation methods. While previously these methods were either too limited in scope (completeness only), crude (eg, augmenting missing data with the mean of the entire dataset or a value’s k-nearest neighbor), or computationally expensive (eg, individual values calculated via regression models based on predetermined sets of correlated features), our review suggests that these tasks are being increasingly automated. Specifically, data attrition contributing to missingness and conformity at the extraction stage may be minimized with AI data extractor algorithms, such as the one described by Lee et al [22]. In cases where further extraction is no longer feasible, the dataset may be augmented by (1) using large language models for extracting structured data available in other formats (eg, laboratory values recorded in the text of media files from outside patient records); (2) incorporating or cross-referencing data from well-established outside data repositories (eg, the US Social Security Death Index for mortality records [2] or the Database of Genotypes and Phenotypes and the Biologic Specimen for biomarkers of heart failure and other conditions [33]); or (3) generating synthetic data, for example, by modeling providers’ behaviors with respect to different information types or sources [3] and by using GANs to create synthetic care episodes based on longitudinal EHR observations [19].

### Data Performance Assessment

Correctness or accuracy was by far the most reported measure among the data performance indicators examined in our review. While certainly integral to assessing a dataset’s usability and potential for downstream clinical or research impact, correctness alone is insufficient to guarantee the success of said applications. A technically “correct” dataset may still be practically limited if it is outdated, biased, inconsistent, or entirely idiosyncratic. We, therefore, recommend that future data assessments consider including additional measures of recency, fairness, stability, and shareability, respectively, among their core set of performance indicators as they each contribute a unique measure of a dataset’s applicability. Importantly, our review noted considerable heterogeneity in the definitions used for these additional measures (eg, by defining data recency in terms of whether the information was logged into the EHR within a set time or whether it represents a patient’s state at a given time period [Table 1] [10,13]), suggesting that further efforts are needed to harmonize outcome definitions in the field of data performance analysis in particular. Nonetheless, the predominance of internal standard comparisons for measuring recency and stability in our review demonstrates that these indicators may be essential for individualized EHR DPAs and should, therefore, be considered on a case-by-case basis (eg, in epidemiology where the timing and consistency of reporting can be of essential importance, or quality improvement initiatives where a researcher might want to compare pre- vs postintervention results). Likewise, shareability ought to be considered in the case of assessing dataset performance for interoperability purposes (eg, with data integrations, sharing, and reporting).

As discussed previously, data fairness assessments can and should be considered for monitoring overall EHR bias, as well as the bias inherent to any data imputation methods as discussed above. Our review points to the fact that this is a rapidly developing field, with fairness assessments to date mostly requiring manual review against national guidelines or disease registries, or, in the case of synthetic data, real EHR datasets [41-43]. Nonetheless, such gold standards are not always readily available (eg, What is the standard distribution of age or race in the real world?), so tech-savvy researchers have more recently resorted to detecting fairness during the validation of machine learning models or algorithms instead of the data itself [41-43]. Several research articles from our analysis proposed ways of automating the process. Fu et al [3] present a straightforward way of measuring the agreement of AI-generated synthetic data against a gold standard dataset. Zhang et al [19] suggest that while such straightforward analysis may be valuable, it is insufficient to measure true fairness, and they go on to propose a method of measuring bias via Jansen-Shannon divergence, which can be calculated for comparisons of real-world and synthetic data. The latter article also suggests a way of preventing synthetic data drift through condition regularization (ie, minimizing contrastive loss by regularizing the synthetic dataset against a real dataset distribution) and fuzzying (ie, adding controlled noise to broaden the dataset distribution before the AI training phase). To our knowledge, this is the most recently proposed technique for fairness assessment in the field. More research is needed to validate and augment the technique. Whether through Jansen-Shannon divergence or alternative methods, we recommend that all future data assessment projects measure and report model performance and fairness for sensitive groups.

Finally, Garcia-a-de-Leon-Chocano et al [24] propose a way of calculating data robustness. The calculation draws on comparing tolerable versus nontolerable issues with DQ, which may be particularly important prior to using the information. We highly suggest that DQ assessments conduct a robustness calculation immediately before calculating data performance measures for downstream applications, which will allow for timely intervention in the case of significant issues with data completeness, conformity, or plausibility that merit additional data collection, review, or imputation steps as discussed above. The above findings and recommendations are summarized in Table 4.

**Table 4.** Recommendations for future EHR^a^ data quality and performance assessments.

Issue	Recommendation
**Article characteristics**	
Paucity of specialty-focused EHR data assessments	Incentivize (eg, through quality improvement initiatives and grants) more EHR data assessments, particularly in psychiatry, emergency medicine, and surgical specialties
Incomplete reporting	Use standardized frameworks for measuring and reporting data quality and performance assessments (eg, Table 1)
Poor replicability	Describe DQA^b^ methods in enough details such that they could be replicated by a research team at a different institution
Limited generalizability	Use already available data quality tools and standards (eg, DQA Guidelines proposed by Weiskopf et al [21]) before developing proprietary methodologies
**DQA**	
Inconsistent methodologies	Analyze completeness, conformance, and plausibility at every DQA (completeness only may be applicable for quick data quality checks)
Data missingness and nonconformity	Use available AI-based data extraction algorithms (eg, Lee et al [22]), and augment data using external and synthetic datasets (eg, Zhang et al [19])
**Data performance assessment**	
Inconsistent methodologies	Augment correctness or accuracy measurement with recency, fairness, stability, and shareability performance metrics
EHR data bias	Automate data fairness assessments by measuring agreement of AI-extracted data against an gold standard dataset (eg, manually extracted data) and preventing drift via condition fuzzying and regularization (eg, Zhang et al [19])
Timeliness of analysis	Calculate dataset robustness prior to detailed data quality and performance analysis (eg, as described by García-de-León-Chocano et al [24])

a EHR: electronic health record.

b DQA: data quality assessment.

### Further Recommendations

Based on the review and our team’s experience with DQ improvement initiatives, we recommend that administrators minimize modifiable DQ and performance issues arising during extraction by first using Internet of Things devices (eg, “smart” patient beds and infusion pumps) that directly upload measurements or settings to the EHR instead of requiring manual data entry. Second, the EHR’s interface should be anchored to a predefined data workflow and ontological structure agreed upon in collaboration with clinical and data administrators (eg, encounters start at the time of patient check-in instead of when a physician first sees the patient, and all encounter times are recorded in 1 location using standard units). Finally, the plausibility of automatically entered data should be periodically validated such that corrections can be made when necessary (eg, a minute-by-minute electrocardiogram plausibility check that can detect if an electrocardiography lead falls off a patient’s chest and needs to be replaced to record accurate measurements). Wherever possible, a reference data format (eg, electrocardiogram voltage between 0.5 and 5 mV) for the validation should be provided.

To minimize modifiable issues arising during data mapping, we furthermore recommend first establishing rules for how to treat (1) “missing,” (2) “modified,” or (3) “overlapping” data, such as whether (1) fields with no value should be regarded as data points or artifacts; (2) data points that have been subsequently modified should be updated or retained; and (3) one data source should take precedence over another in case of duplicate records (eg, weight recordings measured by weighing scale should supersede those measured by a hospital bed). Finally, standards for parent-child encounters should be instituted (eg, if a postoperative outpatient clinic visit should be assigned as a unique encounter or as a child encounter of the parent surgery visit).

The provenance of outside facility records, which can be used to identify potential issues with externally collected data, should also be maintained (eg, keeping records of where and when outside laboratory measures were taken in order to identify potential issues with more or less accurate laboratory techniques).

### Limitations

While this scoping review provides valuable insight into the existing literature on EHR DQ analytics, it has several limitations. Foremost, it is important to acknowledge the limited sample size of 154 articles using our original search criteria, and consequently also the limited number of 26 original research articles which were included in our final analysis after full-text review. Among these articles, there was significant heterogeneity in settings and outcomes of interest, which may limit the validity of direct comparisons between the studies, as well as the generalizability of our findings. The review was furthermore restricted to articles available in the PubMed library, which may introduce a potential publication bias, as well as to articles available only in English, which may introduce a language bias to our study selection and subsequent analysis. Finally, while the review focused on EHR DQ and performance assessments, it did not include adjacent areas that may have a pronounced impact on clinical data recording and use such as EHR implementation or use. Future research should consider broader inclusion criteria and explore additional dimensions of EHR DQ to provide a more comprehensive understanding of this important topic.

### Conclusions

The findings of this scoping review highlight the importance of EHR DQ analysis in ensuring the accuracy and reliability of clinical data. Our review identified a need for specialty-specific data assessment initiatives, particularly in the fields of psychiatry, emergency medicine, and surgery. We additionally identified a need for standardizing DQ reporting to enhance the replicability and generalizability of outcomes in the field. Based on our review of the existing literature, we recommend analyzing DQ in terms of completeness, conformance, and plausibility; data performance in terms of correctness; and use case–specific metrics such as recency, fairness, stability, and shareability. Notably, our review demonstrated several examples of DQ improvement with the use of AI-enhanced data extraction and supplementation techniques. Future efforts in augmenting DQ through AI should make use of data fairness assessments to prevent the introduction of synthetic data bias.

## Supplementary material

10.2196/58130 Multimedia Appendix 1 Search terms.

10.2196/58130 Multimedia Appendix 2 Study characteristics.

10.2196/58130 Checklist 1 PRISMA-ScR (Preferred Reporting Items for Systematic Reviews and Meta-Analyses extension for Scoping Reviews) checklist.

## Abbreviations

AI: artificial intelligence
DPA: data performance assessment
DQ: data quality
DQA: data quality assessment
EHR: electronic health record
GAN: generative adversarial network
MIRACUM: Medical Informatics in Research and Care in University Medicine
PCORnet: The National Patient-Centered Clinical Research Network
PRISMA-ScR: Preferred Reporting Items for Systematic Reviews and Meta-Analyses extension for Scoping Reviews
