# Supplementary material for: Electronic Health Record Data Quality and Performance Assessments: Scoping Review
Source: JMIR Med Inform. 2024 Nov 6;12:e58130. doi: 10.2196/58130 (PMC11559435; doi:10.2196/58130)
Supplement: Multimedia Appendix 1 [file medinform-v12-e58130-s001.docx]

| **Search terms** | **Date Performed** |
| --- | --- |
| "Data Quality Assessment" or "EHR data quality" | May 1, 2023 |
| ("data quality" [tiab] OR "ETL Process" [tiab]) AND "EHR" [tiab] AND ("QA" [tiab] OR "quality assessment" [tiab]) | May 7, 2023 |
| ("data completeness" [tiab] OR "completeness" [tiab] OR "data plausibility" [tiab] OR "plausibility" [tiab] OR "data conformance" [tiab] or "conformance" [tiab]) AND ("EHR" [tiab] OR "electronic health record" [tiab] OR "EMR" [tiab] OR "electronic medical record" [tiab]) AND ("QA" [tiab] OR "quality assessment" [tiab]) | May 7, 2023 |
